# Screening for the high-need population using single institution versus state-wide admissions discharge transfer feed

**DOI:** 10.1186/s12913-023-10017-5

**Published:** 2023-10-17

**Authors:** Francis Salvador Balucan, Benjamin French, Yaping Shi, Sunil Kripalani, Eduard E. Vasilevskis

**Affiliations:** 1 Section of Hospital Medicine, Division of General Internal Medicine and Public Health, Vanderbilt University Medical Center, 2525 West End Ave, Nashville, TN 37212 USA; 2 Department of Biostatistics, Vanderbilt University Medical Center, Nashville, TN USA; 3 Vanderbilt Center for Health Services Research, Nashville, TN USA; 4 Vanderbilt Center for Clinical Quality and Implementation Research, Nashville, TN USA; 5 Geriatric Research Education and Clinical Center, VA Tennessee Valley, Nashville, TN USA

**Keywords:** High need high cost population, Admission discharge transfer feed, Health equity, Digital redlining

## Abstract

**Background:**

Access to programs for high-needs patients depending on single-institution electronic health record data (EHR) carries risks of biased sampling. We investigate a statewide admission, discharge, and transfer feed (ADT) in assessing equity in access to these programs.

**Methods:**

This is a retrospective cross-sectional study. We included high-need patients at Vanderbilt University Medical Center (VUMC) 18 years or older, with at least three emergency visits (ED) or hospitalizations in Tennessee from January 1 to June 30, 2021, including at least one at VUMC. We used the Tennessee ADT database to identify high-need patients with at least one VUMC ED/hospitalization. Then, we compared this population with high-need patients identified using VUMC’s Epic® EHR database. The primary outcome was the sensitivity of VUMC-only criteria for identifying high-need patients compared to the statewide ADT reference standard.

**Results:**

We identified 2549 patients with at least one ED/hospitalization and assessed them as high-need based on the statewide ADT. Of those, 2100 had VUMC-only visits, and 449 had VUMC and non-VUMC visits. VUMC-only visit screening criteria showed high sensitivity (99.1%, 95% CI: 98.7 − 99.5%), showing that the high-needs patients admitted to VUMC infrequently access alternative systems. Results showed no meaningful difference in sensitivity when stratified by patient’s race or insurance.

**Conclusions:**

ADT allows examination for potential selection bias when relying upon single-institution utilization. In VUMC’s high-need patients, there’s minimal selection bias when depending on same-site utilization. Further research must understand how biases vary by site and durability over time.

## Introduction

Access to specialized health programs for high-need patients often depends upon identification with electronic health record (EHR) data based on retrospective costs or utilization. However, the current lack of interoperability [1] between EHRs and delays in accessing claims data hampers the ability to gather medical information to understand the total healthcare utilization of patients [2]. As a result, high-needs patient identification is often based on single-institution EHR data rather than comprehensive utilization across regional or state-wide utilization.

The high-need population is heterogeneous hence understanding comprehensive utilization trajectories is important to understand which sub-populations are amenable to intervention [3]. Incomplete healthcare utilization data may additionally lead to missed opportunities for appropriate care pathways and refers. For example, some may benefit from earlier referral to hospice, while others may only have a transient utilization because of surgery or cancer that calls for rehabilitative support measurers. Finally, identifying high-need patients based on single-institution healthcare utilization carries the risk of biased sampling. For example, hospitals often select high-need patients based on recurrent emergency room and hospitalization visits from their hospital or health system. Patients, however, may seek care in multiple hospitals or health systems due partly to their zip code, preferences, or insurance status. Furthermore, various decisions influence hospital choice, including ambulance transport decisions, hospital bed availability, and severity of illness [4–6]. Although efficient, identifying high-needs patients with single-institution EHR data may unintentionally exclude high-needs patients and potentially worsen disparities in access. This is an example of digital redlining or creating and maintaining technology practices that embed discriminatory practices against marginalized groups [7].

A new regulatory requirement from the Centers for Medicare and Medicaid Services (CMS) requires that hospitals, including behavioral health and critical access hospitals, send real time admission, discharge, and transfer (ADT) event notifications to all providers primarily responsible for a patient’s care [6]. Furthermore, newer organizations are increasingly aggregating these ADT data at the state level and supplying this data feed to participating hospitals. Analysis of newly available ADT records provides an opportunity to understand existing biases that may reflect digital redlining when relying upon single-institution data [8].

Vanderbilt University Medical Center (VUMC) has a hospital program, the Vanderbilt Interdisciplinary Care Program (VICP), that provides consistent and coordinated care for patients with recurrent healthcare utilization. Upon development, the hospital program could only select patients based on the same hospital ED visits and readmissions. With new access to state-wide hospitalization data, our primary objective was to quantify how many patients are excluded from this program using data limited to the same hospital ED visits. Our secondary goal was to understand whether selection patterns based on same-hospital data differed by race or insurance status.

## Methods

### Study population

We performed a retrospective cross-sectional study among all patients 18 years or older admitted to VUMC from January 1 to June 30, 2021, recorded in the VUMC Epic EHR and in the ADT database from the same time. VUMC is part of Vanderbilt Health, a system of clinics and hospitals across middle Tennessee and neighboring states with 1,615 licensed hospital beds at seven hospitals, 141,529 emergency room visits, and 55,969 hospital discharges in fiscal year 2022 [9]. Vanderbilt Health is part of the Vanderbilt Health Affiliated Network Accountable Care Organization encompassing 13 Health Systems and 73 Hospitals with 316,718 attributed patients [10]. The analysis excluded patients if their only admissions were at the Vanderbilt Children’s Hospital, Vanderbilt Psychiatric Hospital, or Vanderbilt Stallworth Rehabilitation Hospital. The institutional review board approved this study as minimal risk and waived informed consent requirements.

### Vanderbilt interdisciplinary care program (VICP) eligibility criteria

VICP is an interdisciplinary, interprofessional team (internal medicine physicians focusing on hospital medicine, advanced practice providers, case managers, social workers, pharmacists, and nurses) providing continuity of coordinated care for high-need, medically and socially complex patients. Patients admitted to the hospital medicine service are screened weekly for eligibility to the program if they had three or more ED visits or hospital admissions in the six months preceding referral to the program as noted only in the VUMC Epic EHR (VICP Criteria).

### Data sources

We used two primary data sources. Through the VUMC Clinical Informatics Core, we extracted data from the VUMC Clarity Enterprise Data Warehouse (EDW), a relational database of data stored in the VUMC Epic EHR, including emergency room, inpatient, and observation admissions. The Tennessee Hospital Association’s Admission/Discharge/Transfer (THA ADT) database was the second data source. When a patient has a hospital or emergency department ADT event within Tennessee, information about that patient’s visit (including, but not limited to, demographic data, information on the source facility, and primary complaint) is packaged into a clinical event notification and sent real-time to the participating hospital. At the time of this analysis, 130 out of 158 hospitals in Tennessee were part of the ADT database [11]. To comply with THA’s data use policy, the researchers can track utilization data only of patients in the ADT database for six months retrospectively and six months prospectively if they had one ED visit or admission at VUMC between January and June 2021.

### Outcome measure

Our primary outcome was the overall sensitivity of current VICP VUMC-EHR screening for the “*High-Need Patient*” based on retrospective 6-month THA ADT data as the reference standard, with at least one of these admissions occurring at VUMC. We used 3 ED visits or hospitalizations in the preceding six months as our definition as it was the institutional definition of high-need during the study. We also aimed to describe the “Underrecognized High-need Patient,” a patient who has had one hospital visit or ED visit at VUMC but had two or more non-VUMC ED or hospital visits.

### Patient demographics

We extracted vital demographic data from the EDW, including age, sex, race, ethnicity, insurance status, and distance in miles from primary residence to VUMC.

### Statistical analysis

We summarized patient demographics and clinic characteristics (hospital admissions, ED visits) using the median (25th, 75th percentile) for quantitative variables and frequency (percentage) for categorical variables.

Enrollment Screening Performance Characteristics. One can think of screening criteria performance as a diagnostic test. In this case, the “true” gold standard high-need patient would be one with three or more ED visits/hospitalizations anywhere in participating Tennessee hospitals during the study period, including at least one at VUMC. The VICP screen-positive high-need patient would have three or more ED visits/hospitalizations at VUMC. This patient would be a true positive based on the VICP screen test. The VICP screen-negative patient would have three or more ED visits/hospitalizations in the ADT data, including at least one ED/hospitalization at VUMC. This patient would have a false negative based on the VICP screen test. Sensitivity is equal to the number of VICP screen-positive patients (true positives) divided by the sum of VICP-screen-positive (true positives) and screen-negative patients (false negatives). Sensitivity (Eq. 1) reflects the ability of VICP’s screening criteria to identify “true” high-need patients as identified with the ADT data. (Table 1). We further stratified results according to race and insurance status to assess for any inequities based on these characteristics. We did not examine ADT data for all VUMC patients with one to two ED/hospitalizations (true negatives) since our study aim was to quantify the number of underrecognized high-need patients.

**Table 1:** Tennessee ADT identification of high need patients, including at least one VUMC ED/Hospitalization (Reference Standard)

**VICP EHR Screening of or High Need Patient**		High Need Patient (At least 3 ED/hospitalizations in Tennessee, including at least one at VUMC	Not a High Need Patient- (1 or 2 ED/hospitalizations in Tennessee, including at least one at VUMC)
	+ (At least 3 ED/hospitalizations at VUMC only)	True Positive	False Positive NA: Data not collected from ADT for patients with < 3 admissions
	(1 or 2 ED/hospitalizations at VUMC only)	False Negative	True Negative – N/A: Data not collected from ADT for patients with < 3 admissions

We used a two-sided 0.05 significance level to define statistical significance. All statistical analyses were performed using R [12] and Hmisc package [13].

## Results

From January 1, 2021, to June 30, 2021, we identified 2549 patients recorded as “high-need” based on the THA ADT as the reference standard, with at least one VUMC ED visit or hospitalization (Table 2). Of the 2549 patients in the THA ADT, 449 had VUMC and non-VUMC visits, and 2100 had only VUMC visits.

The current screening VICP criteria using the VUMC EHR shows high sensitivity (99.1%, 95% CI: 98.7 − 99.5%). The results show that most patients discharged from VUMC get readmitted to VUMC, and high-need patients in the study infrequently access alternative health systems within the region. Lastly, the results show no difference regarding race or insurance (Tables 3 and 4).

Equation 1:

1 $$Sensitivity= \frac{VICP\, Criteria\, High\, Need\, Patient}{Underrecognized\, High\, Need+VICP\, Criteria\, High\, Need)}$$

**Table 2:** Patient Characteristics

	Overall	Underrecognized High-need	VICP VUMC EHR- Criteria High-need Patients
Total Unique Patients	2549	23	2526
**Distance to Hospital (miles)**	33 (11,82)	11 (7, 43)	33 (11, 83)
**Acute Care Utilization**			
VUMC ED Visits	1 (0,2)	1 (1, 2)	1 (0, 2)
VUMC Inpatient/Observation Admissions	2 (1,3)	1 (0, 2)	2 (1, 3)
Non-VUMC ED Visits/Inpatient Observation Admissions	3 (3,4)	4 (2,9)	0 (0,0)
**Race**			
White	2006 (79%)	18 (78%)	1988 (79%)
Black	417 (16%)	5 (22%)	412 (16%)
Other	88 (3.5%)	0 (0%)	114 (4.5%)
Missing	12	0	12
**Ethnicity**			
Not Hispanic	2446 (97%)	22 (96%)	2424 (97%)
Hispanic or Latino	83 (3.3%)	1 (4.3%)	82 (3.3%)
Missing	20	0	20
**Insurance**			
Commercial	907 (36%)	5 (22%)	902 (36%)
Traditional Medicare	674 (26%)	1 (4.3%)	673 (27%)
Medicare Advantage	531 (21%)	8 (35%)	523 (21%)
Medicaid	241 (9.5%)	6 (26%)	235 (9.3%)
Uninsured/Self Pay/Other	115 (4.5%)	3 (13%)	193 (7.6%)

We display the median (25th, 75th percentile) for continuous variables and frequency (percentage) for categorical variables.

**Table 3:** Sensitivity of the VICP Criteria with Regards to Race

	All Patients	White	Black	Other
**VUMC VICP EHR Criteria Positive**	2526	1988	412	114
**VUMC VICP EHR Criteria Negative**	23	18	5	0
**THA ADT Screen**	2549	2006	417	114
Sensitivity (%), 95% CI	99.1 (98.7, 99.5)	99.1 (98.7, 99.5)	98.8 (97.8, 99.8)	100 (100, 100)

**Table 4:** – Sensitivity of the VICP EHR Screen with Regards to Insurance

	All Patients	Commercial Insurance	Medicare / Medicare Advantage	Medicaid	Uninsured/ Self-Pay/ Other
**VUMC VICP EHR Criteria Positive**	2526	902	1196	235	193
**VUMC VICP EHR Criteria Negative**	23	5	9	6	3
**THA ADT Screen**	2549	907	1205	241	196
Sensitivity (%), 95% CI	99.1 (98.7, 99.5)	99.4 (99.0, 99.9)	99.3 (98.8, 99.7)	97.5 (95.5, 99.5)	98.5 (96.8, 100)

## Discussion

In this study, we present a novel use of the Admissions Discharge Transfer feed to evaluate potential biases in single-institution screening for the high-need population for a program that aims to enroll patients with a recent history of high healthcare utilization. Our results show that VUMC’s EHR data from the primary hospital shows high sensitivity in identifying high-need patients. Furthermore, we did not observe any statistically relevant differences in sensitivity across race or insurance status. For this specific institution, it is reassuring that selection criteria to date are not biased. This study demonstrates the value of using state-wide ADT data streams to better characterize a health system’s population and determine whether screening biases may exist that could further exacerbate existing inequities in care delivery. Future studies can evaluate all-payer claims databases to show the high-need population’s true prevalence reliably.

To our knowledge, there are no known studies on bias in selection criteria for the high-need population. Kilaru et al. used Dartmouth’s Hospital Referral Regions (HRR) and Hospital Service Areas (HSA) to examine admission patterns and noted that fewer than half the patients were admitted to the HSAs of residence; however, patients living in populous urban HSAs with multiple large and teaching hospitals, tended to remain in same HSAs for inpatient care [14]. However, within the same HSAs, studies of patients moving from one hospital to another, known colloquially as *doctor shopping*, are limited to patients with substance use disorder [15]. Our results would support the findings that “doctor shopping” is rare. Only recently, all-payer claim databases have become available, which give a more comprehensive view of populations. However, there are challenges with timeliness in the availability of this data [16], which in the high-need population is essential for accurate time enrollment into programs.

Our study was reassuring that the current VUMC electronic medical records screening shows high sensitivity in recognizing the high-need population regardless of race or insurance status. Bias occurs when an algorithm systematically favors one outcome over another [17], and there had been concerns in previous studies of how algorithms were trained to distribute resources based on predicted health costs have prioritized healthier White patients over sicker Black patients because of reduced access to care and tend to use fewer health services [18]. Algorithmic and Clinical Decision Support fairness prevents discrimination involving protected groups such as race, gender, religion, physiologic variability, pre-existing conditions, physical ability, and sexual orientation. Although there is an increased focus on bias evaluation using checklists such as the Prediction Model Risk of Bias Assessment Tool (PROBAST) [19], there is still a lack of agreed standards in evaluating clinical decision support tools and prediction models for a thorough analysis of fairness.

Health systems evaluating programs targeting their high-need population would need to be cautious in the assumption that their EHR is of equal sensitivity as VUMC’s screening for this population, as every health system and every region’s referral patterns are different. University of Chicago’s Comprehensive Care Program [20] and Mount Sinai’s PACT [3] programs are in the top metropolitan statistical areas compared to Nashville [21]. Additionally, in the Nashville metropolitan area in 2020, VUMC’s Emergency Room was the busiest in the Nashville metropolitan area with 79,975 ED [22] encounters compared to the next busiest local hospital with 42,488 encounters [23]. Additionally, the medical center serves as a referral center for the region and beyond, with 14.9% of hospital discharges in 2020 from outside Tennessee and 41% outside the counties surrounding the medical center. These admission characteristics are likely to differ across other regions in the country.

The lack of racial or insurance differences in sensitivity in the current screening may mask existing structural inequalities in the care for high-need patients, as there is no systematic study of the prevalence of the high-need population and the population’s referral patterns within Tennessee. Additionally, there are no studies understanding disparities in access to care for this population, which may affect the identification of the population - as our criterion of high need depends on utilization. Tennessee has the second highest rate of hospital closures in the United States, with 13/16 closures since 2010 in the rural areas [24], and this may explain why 55.9% of VUMC’s discharges are not from the Nashville metropolitan area [22]. However, it is unclear how these closures affect access to care for the high-need population and how many patients cannot get to VUMC because of its distance, especially those living in rural counties in Tennessee.

The study results reassured us that we did not appear to inadvertently perpetuate disparities through our screening algorithms for program eligibility as we strove to use a health equity lens 25,26 in implementing our program. The VICP program currently manually screens the electronic medical record, as there were concerns about ensuring fairness in screening before automating through a clinical decision support system. There are no studies on clinical decision support in screening for the high-need population as previously there is disagreement on its definition of high-need [25]. Only recently has Medicare defined the people using a combination of HCC scores and unplanned admissions in the last year [26], and the program recently updated our criteria. Despite the positive results, we intend to incorporate the ADT feed into our screening, as referral patterns are not static and can change, especially with hospital acquisitions and closures.

## Limitations

We could only identify “high needs” patients who had a relationship with VUMC through a hospital or ED visit between January 1st, 2021, and June 30th, 2021. We cannot see the total population of “high-need” within middle Tennessee (including those from other healthcare systems). Thus, we cannot quantify the total “high-need” people in this catchment area. Additionally, as we had limited our data set from both sources to high-need patients, we cannot calculate the specificity and negative predictive value of our current VIC VUMC EHR Criteria. Lastly, the VICP criteria for the present study do not match Medicare’s definition of high-need, which combines admissions and HCC score data. Creating differences in the number of visits or the time frame for holidays may alter the sensitivity of VUMCs’ current eligibility criteria. Future research should examine potential screening biases with these newly adopted criteria or criteria that shift the time frame or number of visits.

## Conclusion

Understanding EHR-based algorithmic fairness is essential in the high-need population to avoid the potential for digital redlining. We evaluated a novel use of the Admissions/Discharge/Transfer (ADT) feed in evaluating equity in access to the VUMC Interdisciplinary Care program, an interdisciplinary program for high-need patients. The VUMC-only electronic medical screening for high-need patients is sensitive in identifying this population as validated using the ADT data feed. Different health systems have different contexts and an ADT can allow systems to evaluate for algorithmic fairness of patient selection based on healthcare utilization.

## Data Availability

The datasets used and analyzed during the current study are available from the corresponding author upon reasonable request.
